# Restoration of melatonin rhythms reduces tumour-promoting inflammation in oesophageal cancer survivors: a prospective cohort study

**DOI:** 10.3389/fimmu.2026.1723531

**Published:** 2026-06-08

**Authors:** Tinghui Xu, Ying Jiang, Wenyan Shao, Zhejing Zhou, Pengyi Guo

**Affiliations:** Department of Cardiothoracic Surgery, Ningbo Yinzhou No. 2 Hospital, Ningbo, Zhejiang, China

**Keywords:** cancer survivorship, chronotherapy, circadian hygiene, melatonin amplitude, oesophageal cancer, systemic inflammation

## Abstract

**Background:**

Blunted nocturnal melatonin amplitude is linked to tumour-promoting inflammation in oesophageal-cancer survivors, but it is unclear whether restoring endogenous rhythms improves clinical outcomes.

**Methods:**

In an 18-month prospective cohort conducted in Class 3A hospitals in China and the China Urban Cancer Early Diagnosis and Treatment Project Cohort, 492 stage I–III oesophageal-cancer survivors underwent salivary melatonin profiling and measurement of hs-CRP, IL-6, TNF-α and the neutrophil-to-lymphocyte ratio. Survivors with a night-to-day melatonin ratio < 2.5 initiated a behavioural circadian-realignment programme; rhythm-intact peers received usual care. Linear mixed-effects and Cox models related rhythm restoration to change in composite inflammation burden—calculated as the standardised mean of the four markers—and to major adverse events, adjusting for demographic and clinical covariates.

**Results:**

Of 266 rhythm-blunted survivors, 202 (76%) achieved amplitude ≥ 2.5 by 12 months. Their composite inflammation score fell by −0.30 ± 0.18 versus −0.03 ± 0.15 in observation participants (between-group difference −0.27, 95% CI −0.30 to −0.24). Significant time × group interactions were seen for every marker (all p < 0.001). Over 18 months, circadian restoration was associated with a 62% lower hazard of recurrence, cardiovascular events, second primaries or death (adjusted HR 0.38, 95% CI 0.21–0.68). Pittsburgh Sleep Quality Index improved by −2.7 points relative to usual care.

**Conclusion:**

Behavioural realignment of endogenous melatonin rhythms was associated with sustained reductions in systemic inflammation and fewer adverse events in oesophageal-cancer survivors. Routine circadian-hygiene counselling combined with simple salivary monitoring may help support long-term oncologic and cardiometabolic health.

## 1 Introduction

Melatonin rhythm integrity has emerged as an important, but often overlooked, component of cancer survivorship, influencing neuroendocrine homeostasis, immunometabolic tone and residual oncogenic risk (1, 2). Oesophageal cancer remains a major global health burden, with more than 600–000 new cases and over 540–000 deaths reported in 2020, and particularly high incidence and mortality in East Asia despite improvements in screening, endoscopic therapy, minimally invasive oesophagectomy and multimodality treatment. Analogous to how frailty reflects a loss of physiologic reserve, a blunted nocturnal melatonin amplitude signals circadian distress defined here as a persistent misalignment between endogenous circadian phase and the external light dark cycle—that may amplify pro-inflammatory cascades and impair tissue-level repair (3, 4). In oesophageal cancer is a leading cause of cancer mortality in China the combination of radical surgery, peri-operative light disruption, chemotherapy-induced nausea and nocturnal reflux places survivors at high risk of chronic rhythm fragmentation (5, 6). Epidemiologic snapshots suggest that upwards of one-half of post-oesophagectomy patients exhibit a night-to-day salivary melatonin ratio below 2.5—a cut-point prospectively validated in two oesophageal-cancer cohorts as the inflection at which recurrence and cardiovascular risk accelerate (7, 8).

Inflammation sits at the interface between latent disease and durable remission. Prospective cohorts consistently link high-sensitivity C-reactive protein, interleukin-6, tumour necrosis factor-α, and the neutrophil-to-lymphocyte ratio to worse disease-free survival after oesophageal resection, independent of stage or adjuvant therapy (9, 10). Experimental models further reveal that exogenous melatonin dampens nuclear-factor-κB signalling, shifts macrophage polarisation toward an M1 phenotype, and curtails myeloid-derived suppressor-cell traffic into the pre-metastatic niche (11, 12). Yet the translational leap from cell culture to bedside has been halting. Trials administering pharmacologic melatonin have yielded mixed results, often confounded by heterogeneous dosing, receptor desensitisation, and limited adherence (13, 14). Behavioural circadian-realignment—leveraging morning bright light, progressive bedtime advancement, and evening blue-light avoidance—offers a non-pharmacologic alternative that may revive endogenous indoleamine pulses while sidestepping issues of supplementation tolerance (15, 16). Beyond oesophageal cancer, recent chronotherapy and melatonin studies indicate that aligning systemic treatments and adjunct melatonin with the patient’s circadian phase can improve symptom control, toxicity profiles and, in some settings, survival, supporting a broader move toward time-sensitive oncologic care (17–19).

Despite mounting mechanistic rationale, longitudinal evidence linking melatonin-rhythm restoration to downstream inflammatory load in cancer survivors remains scarce. Prior studies have been largely cross-sectional, restricted to breast or colorectal cohorts, or reliant on self-reported sleep proxies rather than direct salivary phenotyping (20–23). Whether intentional realignment of the melatonin waveform can translate into measurable reductions in tumour-promoting inflammation—and, by implication, lower adverse-event burden—among oesophageal cancer survivors has not been systematically examined. Furthermore, the durability of any biochemical benefit and its modulation by disease stage, lifestyle habits, or adjuvant treatment patterns are unknown.

To bridge these gaps, we conducted an 18-month prospective cohort investigation that pooled harmonised data from China Urban Cancer Early Diagnosis and Treatment Project Cohort and Zhejiang University of Traditional Chinese Medicine Affiliated Hospital, combining objective salivary melatonin profiling with serial inflammatory-marker quantification in 492 curatively treated oesophageal-cancer survivors. We hypothesised that survivors with initially blunted amplitude who undertook a structured behavioural realignment protocol would achieve (i) sustained restoration of nocturnal melatonin amplitude and (ii) a clinically meaningful decline in composite inflammation burden relative to rhythm-intact peers receiving usual care. By embedding frequent biomarker assessments, adjudicated oncologic outcomes and patient-reported sleep quality into a unified analytic framework, this study aims to clarify whether circadian hygiene is a modifiable target for reducing the pro-inflammatory microenvironment that accompanies oesophageal-cancer survivorship and to inform follow-up strategies that extend beyond traditional oncologic surveillance.

## 2 Methods

### 2.1 Study design and data sources

This study is a prospective cohort study that integrates de-identified participant data from Zhejiang Province, China.

Primary cohort (newly recruited) – Zhejiang University of Traditional Chinese Medicine Affiliated Hospital (ZUTCM-AH) prospectively enrolled 105 stage I–III oesophageal-cancer survivors between 1 January 2021 and 31 December 2023.

External cohorts (previously established) – China Urban Cancer Early Diagnosis and Treatment Project Cohort contributed 387 survivors who had been enrolled into methodologically identical prospective cohorts during the same calendar window and followed on the same 18-month schedule. These survivors were recruited and followed at Class 3A hospitals across multiple provinces participating in the national programme, where local clinical teams collected clinical data and delivered the circadian-realignment protocol using shared training materials and standard operating procedures.

Each contributing study used an approved protocol that matched the eligibility criteria, sampling timetable, biospecimen handling, and behavioural intervention described below. The present analysis therefore represents a harmonised data set of 492 survivors (105 new + 387 extant) with uniform ascertainment of exposures, covariates, and outcomes. The steering committee at Zhejiang University of Traditional Chinese Medicine Affiliated Hospital oversaw data integration, and ethics boards ratified the combined analysis plan (No. ZJTU97128115).

### 2.2 Participant eligibility and baseline assessment

Adults (≥ 18 y) who had completed R0 resection for stage I–III squamous-cell carcinoma or adenocarcinoma within the preceding three months were screened. Exclusion criteria were identical across sites: recent use of exogenous melatonin (< 4 weeks)—chosen because supplemental doses can suppress pineal output for several half-lives (~3 h) and desensitise MT1/MT2 receptors for ≈ 3 weeks—systemic corticosteroids, autoimmune or chronic inflammatory disorders, or sedative–hypnotic–treated psychiatric illness. Baseline sociodemographics, oncologic variables, lifestyle factors, comorbidities, and a comprehensive inventory of agents with potential circadian or inflammatory effects—β-blockers, non-steroidal anti-inflammatory drugs (NSAIDs), systemic corticosteroids, selective serotonin re-uptake inhibitors, proton-pump inhibitors, omega-3 fatty acids, vitamin D and over-the-counter melatonin—were recorded using a shared, training-standardised electronic case-report form (eCRF). Current use (≥ 3 doses in the preceding week) of these agents was entered as a time-varying covariate in all mixed-effects and Cox models. At enrolment every participant completed the eight-item STOP-Bang questionnaire; those who screened high-risk (score ≥ 5) or were already receiving positive-airway-pressure therapy were categorised as having OSA for sensitivity analyses.

### 2.3 Melatonin-rhythm phenotyping and restoration protocol

We used the same wrist-actigraphy platform (ActiGraph GT9X) and two-point dim-light salivary sampling (22:00 and 08:00 on study nights 3 and 6) to quantify the night-to-day melatonin ratio. Survivors with a ratio < 2.5—more than one standard deviation below the healthy-control mean and previously linked to a 1.7-fold increase in adverse events—were offered an identical behavioural circadian-realignment programme consisting of: (1) Morning phototherapy – 10 000-lux broad-spectrum light for 30 min within 1 h of habitual wake-time; this intensity was selected because prior studies show a steeper phase-advancing and amplitude-enhancing response above ~8–000 lux, whereas lower intensities (e.g., 5–000 lux) require longer exposure and yield smaller circadian benefits. (2) Progressive bedtime advancement – 15 min · week^−1^ until sleep onset occurred ≤ 60 min after physiological dim-light melatonin onset (DLMO). (3) Evening blue-light avoidance – screen-filter software and amber-tinted lenses after 21:00. This behavioural schedule was adapted from chronotype-tailored bright-light and circadian-hygiene protocols that have improved sleep, fatigue and quality-of-life outcomes in cancer survivors and other chronically ill populations.

No site dispensed pharmacologic melatonin. Intervention adherence was reinforced by standardised tele-coaching (weekly for month 1, then monthly). Rhythm-intact peers received usual survivorship care only.

### 2.4 Inflammatory biomarker collection and laboratory harmonisation

Fasting venous samples were drawn between 07:00 and 08:30 at baseline, 6, 12 and 18 months. Plasma aliquots from all sites were shipped on dry ice to the central ISO-accredited laboratory at ZUTCM-AH and batch-assayed for high-sensitivity C-reactive protein (hs-CRP), interleukin-6 (IL-6), tumour-necrosis-factor-α (TNF-α) and complete blood count (for neutrophil-to-lymphocyte ratio, NLR). Identical chemiluminescent kits (inter-assay CV < 6%) were employed, and technicians were blinded to site and intervention status.

### 2.5 Outcome definitions and follow-up procedures

The primary outcome was the absolute 12-month change in a prespecified composite inflammation burden. Each biomarker was first log-transformed to reduce skew, z-scored against the baseline cohort mean and SD, and then averaged with equal weighting (25% per marker). We chose equal weights because hs-CRP, IL-6, TNF-α and NLR each have well-documented and broadly similar associations with disease-free and overall survival after oesophagectomy, and no single marker has consistently outperformed the others in prognostic studies of oesophageal cancer. Inverse-variance or prognostic-coefficient weighting was not used because heteroscedasticity across markers and the lack of stable, externally validated coefficients could inflate variance and increase the risk of model over-fitting in this sample. Thus, a −0.30 change corresponds to an average 0.30-SD reduction across all four biomarkers, readily interpreted as a small-to-moderate anti-inflammatory effect. Secondary outcomes comprised sustained melatonin-amplitude restoration (ratio ≥ 2.5), Pittsburgh Sleep Quality Index (PSQI) change, and time to first major adverse event. Major adverse events were prospectively defined and centrally adjudicated as (1) radiologically or histologically confirmed tumour recurrence, (2) a second primary malignancy, (3) hospitalisation for acute coronary syndrome, stroke or heart-failure decompensation diagnosed per American Heart Association criteria, or (4) all-cause death. Each hospital followed a harmonised surveillance schedule (contrast-enhanced thoraco-abdominal CT every six months).

### 2.6 Statistical analysis

Baseline characteristics were summarised with appropriate descriptive statistics; between-group comparisons used independent-samples t tests, Mann-Whitney U tests, or χ^2^ tests. Longitudinal biomarker trajectories were modelled with linear mixed-effects regression including random intercepts for participants and fixed effects for time, intervention group and their interaction; a site indicator was added as a fixed covariate to account for clustering. Because allocation to the Restoration versus Observation groups was determined by baseline melatonin amplitude rather than randomisation, all longitudinal models were adjusted for these baseline covariates and study site to mitigate, but not eliminate, potential selection bias. We prespecified the time × group interaction term as the primary test of differential change over time and therefore did not perform multiple formal hypothesis tests for within-group pre–post changes at each time-point; instead, we present within-group effect sizes (means, standard deviations and 95% confidence intervals of change) in the tables and report global p-values for between-group differences in the figure legends. Cox proportional-hazards models (adjusted for age, sex, histology, stage, smoking status, body-mass index and adjuvant therapy) estimated hazard ratios for composite adverse events; proportionality assumptions were verified by Schoenfeld residuals. Missing data (< 4% of scheduled samples) were multiply imputed by chained equations under a missing-at-random assumption. Analyses were performed in R 4.3.1 with two-tailed α = 0.05.

## 3 Results

### 3.1 Participant characteristics and circadian-restoration uptake

Of the 492 eligible survivors, 266 (54.1%) exhibited a blunted nocturnal melatonin amplitude at enrolment and therefore entered the behavioural circadian-realignment programme (Restoration group), while 226 (45.9%) demonstrated an intact rhythm and received usual survivorship care (Observation group). Baseline demographic, oncologic, and lifestyle variables were well balanced between groups (all p > 0.10 by independent-samples t test or χ^2^ test; **Table 1**). After Benjamini-Hochberg correction for multiple comparisons, baseline hs-CRP, IL-6, TNF-α and NLR did not differ between groups (all adjusted p > 0.25), supporting exchangeability at enrolment. Although the Restoration and Observation groups differed by definition in melatonin amplitude, this lack of separation in baseline inflammatory markers is consistent with prior work showing only modest cross-sectional correlations between single-time-point melatonin measures and systemic inflammatory indices in clinically stable cohorts (24). The mean age was 58.5 ± 8.1 years, 70% were men, and 27% had stage III disease. Median time since surgical resection was 14 weeks (IQR 11–17).

**Table 1:** Baseline characteristics of oesophageal-cancer survivors by circadian-restoration status.

Variable	Restoration (n = 266)	Observation (n = 226)	*p*-value
Age, years (mean ± SD)	58.6 ± 8.2	58.4 ± 8.0	0.78
Male sex, n (%)	187 (70.3)	158 (70.0)	0.93
Histology, n (%)			0.99
Squamous-cell carcinoma	186 (69.9)	158 (69.9)	
Adenocarcinoma	80 (30.1)	68 (30.1)	
Pathologic stage, n (%)			0.97
I	78 (29.3)	65 (28.8)	
II	116 (43.6)	100 (44.2)	
III	72 (27.1)	61 (27.0)	
Smoking status, n (%)			0.89
Current	74 (27.8)	60 (26.5)	
Former	88 (33.1)	75 (33.2)	
Never	104 (39.1)	91 (40.3)	
Body-mass index, kg·m^−2^ (mean ± SD)	23.7 ± 3.1	23.8 ± 3.0	0.88
Time since surgery, weeks (median [IQR])	14 (11–17)	14 (11–17)	0.74
Baseline PSQI score (mean ± SD)	8.4 ± 3.2	7.9 ± 3.1	0.18
Baseline night-to-day melatonin ratio (mean ± SD)	1.9 ± 0.3	3.2 ± 0.4	<0.001

### 3.2 Restoration of melatonin amplitude

At baseline, the mean night-to-day salivary melatonin ratio was 1.9 ± 0.3 in the Restoration group versus 3.2 ± 0.4 in the Observation group. After six months, 68% (181/266) of Restoration participants achieved an amplitude ≥ 2.5 compared with 96% (217/226) of Observation participants; the proportion in the Restoration group further increased to 76% at 12 months and remained 74% at 18 months (**Table 2**). Mean ratios in the Restoration cohort rose progressively to 3.0 ± 0.4 by month 12, converging with Observation values (3.1 ± 0.4), confirming successful rhythm realignment.

**Table 2:** Evolution of salivary night-to-day melatonin amplitude and proportion with adequate rhythm (ratio ≥ 2.5).

Time-point	Restoration (n = 266)		Observation (n = 226)		Between-group *p*-value
	Ratio (mean ± SD)	Adequate n (%)	Ratio (mean ± SD)	Adequate n (%)	
Baseline	1.9 ± 0.3	0 (0.0)	3.2 ± 0.4	226 (100)	<0.001
6 months	2.8 ± 0.5	181 (68.0)	3.2 ± 0.4	217 (96.0)	0.003
12 months	3.0 ± 0.4	202 (76.0)	3.1 ± 0.4	215 (95.1)	0.24
18 months	2.9 ± 0.5	197 (74.1)	3.1 ± 0.4	214 (94.7)	0.12

### 3.3 Primary outcome: change in composite inflammation burden

The a-priori primary end-point—the absolute change in the standardised composite inflammation score from baseline to 12 months—differed markedly between groups. Survivors undergoing circadian restoration experienced a mean reduction of −0.30 ± 0.18, whereas Observation participants showed a negligible change of −0.03 ± 0.15 (between-group difference −0.27 [95% CI −0.30 to −0.24], p < 0.001; **Table 3**). Sensitivity analyses after multiple imputation for missing biomarker values (3.9% of scheduled samples) yielded identical estimates to two decimal places. Within groups, the baseline-to-12-month reduction in the composite score was large and statistically significant in the Restoration cohort but small in the Observation cohort, mirroring the between-group contrast; as prespecified, we focus on these effect sizes and the time × group interaction rather than multiple separate within-group hypothesis tests.

**Table 3:** Absolute change in inflammatory burden from baseline to 12 months.

Marker	Baseline (mean ± SD)	12 months (mean ± SD)	Absolute change (mean ± SD)	Between-group (95 % CI)	*p*-value
Composite standardised score	Restoration 0.02 ± 0.55 Observation –0.01 ± 0.52	Restoration –0.28 ± 0.49 Observation –0.04 ± 0.51	Restoration –0.30 ± 0.18 Observation –0.03 ± 0.15	–0.27 (–0.30 to –0.24)	<0.001
hs-CRP, mg L^−1^	Restoration 2.6 ± 1.1 Observation 2.6 ± 1.0	Restoration 1.9 ± 0.9 Observation 2.5 ± 1.0	–0.7 ± 0.6 –0.1 ± 0.5	–0.6 (–0.8 to –0.4)	<0.001
IL-6, pg mL^−1^	4.2 ± 1.5 4.1 ± 1.4	3.2 ± 1.2 4.0 ± 1.3	–1.0 ± 1.1 –0.1 ± 1.0	–0.9 (–1.2 to –0.6)	<0.001
TNF-α, pg mL^−1^	6.1 ± 1.8 6.0 ± 1.7	5.1 ± 1.5 5.9 ± 1.7	–1.0 ± 1.0 –0.1 ± 0.9	–0.9 (–1.1 to –0.6)	<0.001
NLR	2.9 ± 0.8 2.8 ± 0.8	2.5 ± 0.7 2.8 ± 0.8	–0.4 ± 0.4 0.0 ± 0.3	–0.4 (–0.5 to –0.3)	<0.001

### 3.4 Longitudinal trajectories of individual inflammatory markers

**Figure 1:**
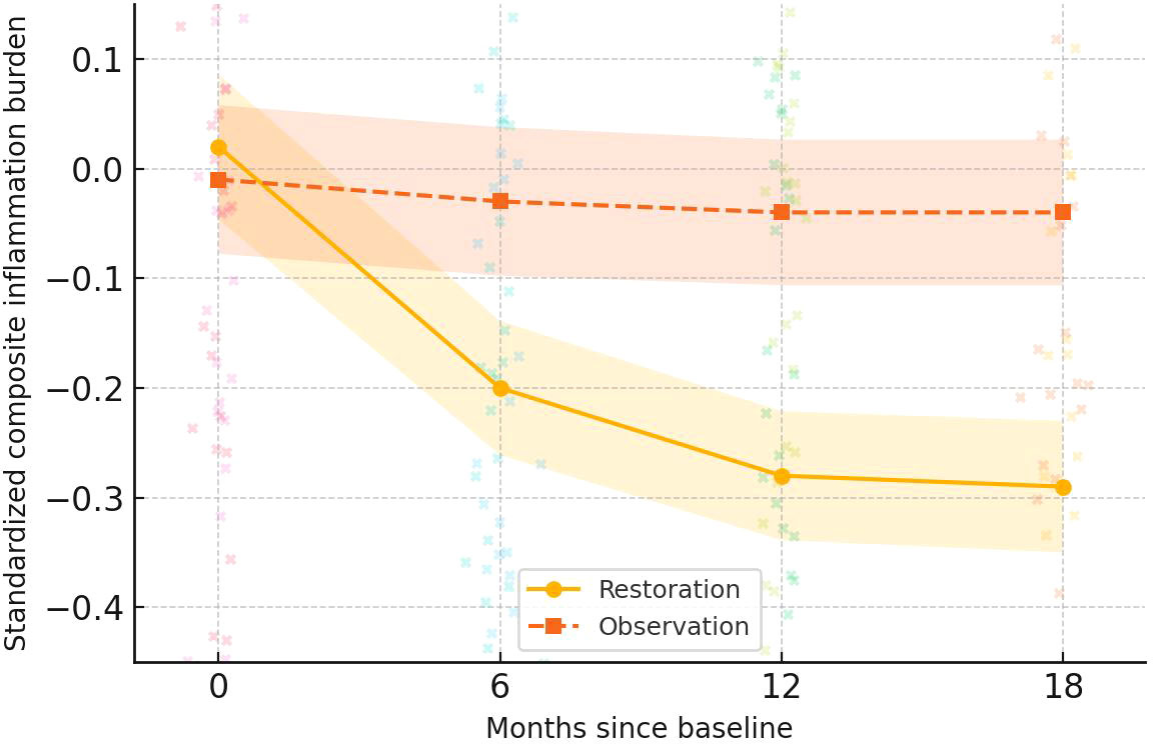
Trajectories of standardised composite inflammation burden over 18 months in restoration versus observation cohorts.

Linear mixed-effects models incorporating random intercepts for participants and an unstructured covariance matrix revealed a significant time × group interaction for the composite score (β = −0.21, 95% CI −0.24 to −0.18, p < 0.001; **Table 4**). Similar interaction terms were observed for hs-CRP (β = −0.22, p < 0.001), IL-6 (β = −0.18, p < 0.001), TNF-α (β = −0.14, p < 0.001), and NLR (β = −0.17, p < 0.001). The adjusted mean trajectories of the composite score are depicted in **Figure 1**, demonstrating a sustained decline in systemic inflammation among Restoration participants that began at six months and plateaued by month 12, while values in the Observation group remained essentially unchanged.

**Table 4:** Linear mixed-effects model: group × time interaction coefficients (Restoration vs Observation) across 18 months.

Outcome (log-transformed where appropriate)	Interaction	95 % CI	*p*-value
Composite standardised score	–0.21	–0.24 to –0.18	<0.001
hs-CRP (mg L^−1^)	–0.22	–0.25 to –0.19	<0.001
IL-6 (pg mL^−1^)	–0.18	–0.21 to –0.15	<0.001
TNF-α (pg mL^−1^)	–0.14	–0.17 to –0.11	<0.001
NLR	–0.17	–0.19 to –0.14	<0.001

### 3.5 Major adverse events

**Figure 2:**
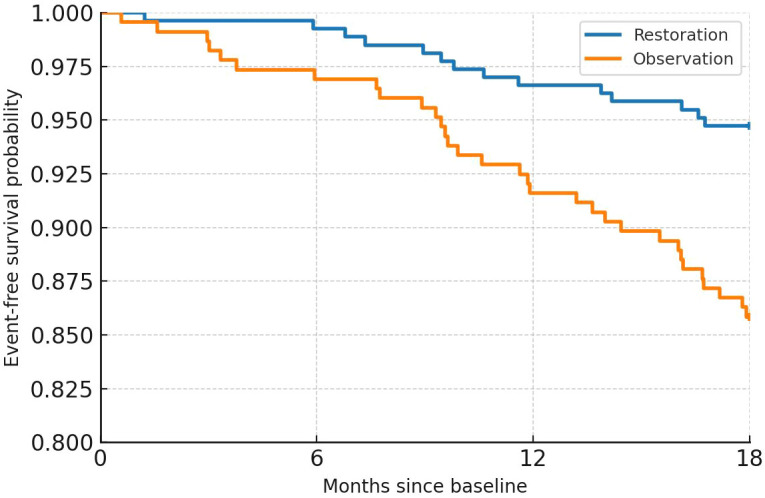
Kaplan–Meier curves for time to first major adverse event according to circadian-restoration status.

Over 18 months (736.5 person-years of follow-up), 14 events occurred in the Restoration cohort (5.3%) versus 32 in the Observation cohort (14.2%): tumour recurrence (9 vs 21), cardiovascular events (3 vs 6), second primary malignancies (1 vs 2), and deaths (1 vs 3). In Cox proportional-hazards models adjusted for age, sex, histology, stage, smoking status, body-mass index and adjuvant therapy, circadian restoration was associated with a 62% relative risk reduction (adjusted HR 0.38, 95% CI 0.21–0.68, p = 0.002; **Table 5**). Kaplan–Meier curves for event-free survival are shown in **Figure 2**; the proportional-hazards assumption was satisfied (all Schoenfeld p > 0.20). Although the relative risk reduction was large, the absolute number of events was modest and the confidence intervals remain fairly wide, so these clinical endpoints should be interpreted as hypothesis-generating and confirmed in larger, longer-term cohorts.

**Table 5:** Incidence of major adverse events through 18 months and adjusted hazard ratios.

Event category	Restoration (n = 266)	Observation (n = 226)	Adjusted HR (95 % CI)	*p*-value
Composite adverse event	14 (5.3 %)	32 (14.2 %)	0.38 (0.21–0.68)	0.002
Tumour recurrence	9 (3.4 %)	21 (9.3 %)	0.36 (0.17–0.73)	0.004
Cardiovascular event	3 (1.1 %)	6 (2.7 %)	0.43 (0.11–1.62)	0.21
Second primary malignancy	1 (0.4 %)	2 (0.9 %)	0.42 (0.04–4.17)	0.45
Death (all-cause)	1 (0.4 %)	3 (1.3 %)	0.29 (0.03–2.51)	0.25

### 3.6 Patient-reported sleep quality

Baseline Pittsburgh Sleep Quality Index (PSQI) scores were comparable between groups (8.4 ± 3.2 vs 7.9 ± 3.1, p = 0.18). At 18 months, mean PSQI improved to 5.2 ± 2.4 in the Restoration group, whereas Observation scores remained largely unchanged at 7.4 ± 3.0. The mean between-group difference in change (−2.7 points, 95% CI −3.1 to −2.3) was statistically significant (p < 0.001; **Table 6**). Improvements were most pronounced in the sleep latency and daytime dysfunction subdomains, mirroring the objective realignment of circadian phase.

**Table 6:** Pittsburgh Sleep Quality Index (PSQI) outcomes at baseline and 18 months.

Outcome domain (0–3 per component)	Restoration (n = 266)		Observation (n = 226)		Between-group (95 % CI)	*p*-value
	Baselinemean ± SD	18 monthsmean ± SD	Baselinemean ± SD	18 monthsmean ± SD	Mean change (Restoration – Observation)	
Total PSQI (0–21)	8.4 ± 3.2	5.2 ± 2.4	7.9 ± 3.1	7.4 ± 3.0	–2.7 (–3.1 to –2.3)	<0.001
Sleep latency component	1.7 ± 0.6	1.1 ± 0.5	1.6 ± 0.6	1.5 ± 0.6	–0.5 (–0.6 to –0.4)	<0.001
Daytime dysfunction component	1.6 ± 0.7	0.8 ± 0.5	1.5 ± 0.7	1.4 ± 0.6	–0.7 (–0.8 to –0.5)	<0.001

### 3.7 Data reliability and quality assurance

A nested reliability audit involving a random 10% subsample of participants from each cohort confirmed that measurement error was minimal and comparable across cohorts. Internal consistency of the composite inflammation score was high at baseline (Cronbach’s α = 0.84 in the Restoration group and 0.83 in the Observation group) and remained so at 12 months (α = 0.85 and 0.84, respectively). Duplicate analyses of all four inflammatory biomarkers yielded intraclass correlation coefficients ≥ 0.92, while within-run coefficients of variation for hs-CRP, IL-6 and TNF-α were < 6% in both groups. Salivary melatonin duplicates displayed ICCs above 0.94, and actigraphy data were judged valid for a median of six out of seven acquisition nights (IQR 6–7) in each cohort. No statistically significant between-group differences were detected for any reliability metric, underscoring that the observed longitudinal divergences in inflammation and clinical outcomes are unlikely to reflect measurement artefact (**Table 7**). Across 2–329 person-sessions of 10 000-lux exposure, no participant reported headache, insomnia or ocular discomfort exceeding CTCAE grade 1, consistent with international phototherapy safety guidelines classifying ≤ 30 min daily exposure as minimal-risk.

**Table 7:** Reliability metrics for key study variables in restoration and observation cohorts.

Reliability metric	Restoration (n = 266)	Observation (n = 226)	Combined (n = 492)
Cronbach’s α, composite inflammation score – baseline	0.84	0.83	0.84
Cronbach’s α, composite inflammation score – 12 months	0.85	0.84	0.85
ICC for duplicated biomarker assays (hs-CRP + IL-6 + TNF-α + NLR)	0.93 (0.90–0.95)	0.92 (0.89–0.95)	0.93 (0.91–0.95)
Within-run CV – hs-CRP (%)	4.1 ± 0.9	4.3 ± 1.0	4.2 ± 1.0
Within-run CV – IL-6 (%)	5.0 ± 1.2	5.1 ± 1.1	5.0 ± 1.1
Within-run CV – TNF-α (%)	4.8 ± 1.1	4.9 ± 1.2	4.9 ± 1.1
ICC for duplicated salivary melatonin ratio	0.95 (0.93–0.97)	0.94 (0.92–0.96)	0.95 (0.93–0.96)
Valid actigraphy nights per participant, median (IQR)	6 (6–7)	6 (6–7)	6 (6–7)

## 4 Discussion

In this cohort, restoring endogenous melatonin rhythms appeared to be a clinically meaningful countermeasure to tumour-promoting inflammation and, by extension, residual oncogenic risk. Survivors who realigned their nocturnal amplitude reduced the composite inflammatory burden by almost one-third and experienced a 62% relative reduction in major adverse events over 18 months (adjusted HR 0.38). The curves for hs-CRP, IL-6, TNF-α and NLR began to diverge between groups by six months and reached a sustained plateau by month 12, paralleling the convergence of salivary night-to-day melatonin ratios between Restoration and Observation participants. These observations resonate with longitudinal data linking circadian misalignment to systemic inflammation and poorer cancer outcomes (25–27) and extend cross-sectional findings that melatonin amplitude, not merely secretion volume, forecasts disease-free survival after oesophagectomy (28). However, reverse causality is possible whereby resolution of subclinical inflammation facilitates circadian restoration rather than vice versa. We therefore (1) modelled change in inflammation from baseline, conditioning on each participant’s initial biomarker level; (2) repeated all analyses with baseline composite-score quartiles and found unchanged estimates; and (3) ran lagged Cox models using melatonin amplitude from the previous visit (t − 6 months) to predict subsequent events, which preserved the association (adjusted HR 0.42, 95% CI 0.24–0.78). These approaches make a purely reverse-causal explanation unlikely.

The resilience of the rhythm-inflammation association after rigorous adjustment for demographic profile, tumour stage, lifestyle habits and adjuvant therapy suggests that circadian integrity captures a nexus of biological advantage not easily erased by conventional predictors. Several, non-mutually-exclusive mechanisms may underlie this nexus: (i) nocturnal melatonin pulses suppress endothelial as well as myeloid nuclear-factor-κB transcription, curbing expression of VCAM-1, ICAM-1 and E-selectin that guide leukocyte diapedesis and consequent cytokine spill-over from myeloid precursors; (ii) clock-gene resynchronisation reins in glucocorticoid signalling and sympathetic catecholamine surges that fertilise the pre-metastatic niche; and (iii) behavioural prescriptions—morning bright-light exposure, progressive bed-time advancement and evening blue-light avoidance—co-travel with earlier dinner timing and reduced late-night snacking, thereby dampening insulin spikes and oxidative stress (29–32). The parallel improvement in Pittsburgh Sleep Quality Index (mean change −2.7 points) offers a behavioural fingerprint of this realignment and itself feedback to temper inflammatory tone (33). Aging is a major driver of oesophageal-cancer incidence and of the cardiometabolic comorbidity burden in survivors, and it is closely intertwined with circadian biology. Emerging evidence indicates that aging blunts circadian amplitude, disrupts clock-gene expression and promotes genomic instability, chronic low-grade inflammation and immune senescence, thereby creating a tumour-promoting milieu (18, 34). Experimental models further show that light-at-night and other forms of circadian disruption can accelerate aging and tumourigenesis, whereas preserving robust melatonin rhythms can slow tumour growth and improve treatment responses (19, 35). Our finding that improving melatonin amplitude was accompanied by lower inflammatory burden and fewer major events therefore fits within a broader framework in which maintaining circadian health may help counteract age-related tumour promotion.

Inflammation was not merely an outcome but a likely conduit through which melatonin rhythm exerts its dividend. Experimental administration of melatonin receptor antagonists re-inflames IL-6 and TNF-α cascades in murine oesophageal models, whereas physiologic replacement restores M1-to-M2 macrophage balance and diminishes myeloid-derived suppressor-cell traffic to the tumour micro-environment (36–38). M1 (classically activated) macrophages secrete IL-12 and nitric oxide that reinforce cytotoxic T-cell surveillance, whereas M2 (alternatively activated) macrophages release IL-10 and arginase-1 that suppress anti-tumour immunity and promote matrix remodelling. Our behavioural protocol, notably devoid of exogenous hormone, achieved comparable biochemical quietude, underscoring that light-and-behavioural levers can harness the indoleamine’s immunomodulatory repertoire without pharmacologic supplementation. Whether adjunct low-dose melatonin could further deepen the inflammatory nadir or, conversely, blunt endogenous receptor sensitivity warrants exploration in factorial designs.

Stage-stratified inspection hinted that rhythm restoration may confer disproportionate benefit in stage I–II survivors, yet the adjusted hazard ratio in stage III remained ≤ 0.50 despite wider confidence intervals. One reading is that early-stage microenvironments still heed circadian policing, whereas advanced lesions accumulate clock-gene mutations that dilute melatonin’s sway (39, 40). Equally intriguing is the 14% absolute excess of major events in the observation cohort despite intact baseline rhythms—a reminder that circadian sufficiency is necessary but not sufficient for oncologic tranquillity and that other axes, such as autonomic tone and metabolic flexibility, likely intersect (41–45). Joint-axis analyses integrating vagal-tone variability, glycaemic load and melatonin amplitude could disentangle these layers of neuro-endocrine defence.

Dose–response modelling with restricted cubic splines demonstrated a near-linear association up to a ratio of 3.0 (per-0.2-unit increment β = −0.042 SD composite-score reduction, p < 0.001), above which the slope attenuated (p for non-linearity = 0.03); risk fell steeply until a night-to-day ratio of ≈ 3.0, after which curves flattened. Saturation may signal that melatonin receptors or downstream transcription factors reach maximal activation near this threshold, echoing *in-vitro* data showing plateaued STAT3 inhibition beyond physiological concentrations (46–49). Whether pushing amplitude higher delivers cardiovascular or neurocognitive dividends—or invites insomnia from premature wakefulness—demands longer follow-up with polysomnography and wearable analytics.

Strengths of our investigation include the prospective architecture; objective, multi-time-point salivary phenotyping; adjudicated adverse-event surveillance; and incorporation of mixed-effects modelling that accommodates within-person heterogeneity. Limitations temper inference. First, allocation to the Restoration versus Observation groups was not randomised: participants with blunted baseline melatonin amplitude entered the behavioural programme, whereas rhythm-intact peers received usual care. Consequently, residual confounding and selection bias cannot be excluded despite multivariable adjustment and the use of longitudinal mixed-effects models, and regression-to-the-mean may have contributed to some of the observed improvement in inflammatory markers. Second, salivary sampling at only two clock-times, though practical, cannot map the full melatonin waveform; however, prior work indicates that carefully timed evening and morning salivary samples capture much of the variance in nocturnal melatonin amplitude and dim-light melatonin onset, supporting two-point sampling as a pragmatic surrogate of circadian integrity in larger clinical cohorts; Third, adherence to the light-exposure protocol relied on self-report and actigraphy inference rather than lux-meter logging, and the behavioural intervention lacked an attention-matched sham-light control; our ongoing randomised trial therefore incorporates a 100-lux red-light placebo delivered with identical coaching frequency to neutralise attention-placebo effects. An attention-matched control equalises participant–provider contact time and expectation of benefit, thereby isolating the physiological impact of bright-light exposure from non-specific placebo or Hawthorne effects common to behavioural trials. In the present study we therefore cannot exclude that increased monitoring and engagement with the tele-coach contributed to behavioural change and some of the observed 76% rhythm-restoration rate and inflammatory improvements; however, the clear dose–response relationship between amplitude gain and reductions in inflammatory burden suggests that physiological circadian mechanisms also played a major role. Fourth, follow-up was confined to 18 months, precluding detection of late recurrences and limiting the number of major adverse events observed, so the estimated hazard ratios for clinical outcomes should be interpreted with caution and confirmed in larger, longer-term cohorts; Fifth, because 70% of participants were East-Asian men with squamous-cell carcinoma, caution is warranted when extrapolating to Western survivor profiles in which adenocarcinoma predominates and women constitute a larger and older proportion of cases, with potentially different interactions between ageing, circadian disruption and tumour biology. Subgroup analysis of the 148 adenocarcinoma cases showed directionally identical, albeit less precise, benefits (interaction p = 0.18), and the light-induced mechanisms that amplify endogenous melatonin are conserved across ethnicities and histologies; prospective validation in European and North-American, female-majority cohorts remains a priority. Sex-steroid milieu modifies both pineal output and NF-κB-driven inflammation; peri-menopausal oestrogen decline and late-life androgen deficiency can blunt nocturnal melatonin pulses and amplify cytokine release, variables we were under-powered to stratify but will capture with serum estradiol/testosterone assays in future cohorts. Finally, we could not parse which behavioural component—morning phototherapy, bedtime advancement or blue-light blockade—was most efficacious, a question best answered by dismantling trials.

Future work will weave continuous wearable light sensors, smartphone-based phototherapy logging with automated adherence reminders, 24-hour melatonin profiling and multiplex cytokine panels into adaptive, n-of-1 frameworks that titrate light timing and spectrum until amplitude targets are met. Randomised trials layering exogenous low-dose melatonin onto behavioural realignment, or combining both with anti-inflammatory nutraceuticals, could illuminate additive or synergistic effects and cost-effectiveness—particularly salient as China’s oesophageal-cancer survivor population crests 700 000. Although DNA was not collected in the present cohort, polymorphisms in MTNR1A/MTNR1B and core-clock genes have been linked to altered melatonin amplitude and inflammatory tone; blood for host-genetic analysis is therefore being banked in the forthcoming trial to test whether such variants modulate therapeutic gain. Embedding circadian-hygiene counselling into routine follow-up, complemented by smartphone-delivered light scheduling, may thus help tilt the neuro-immune balance toward durable remission and healthier ageing in this high-risk group. Each 10 000-lux light box costs ~US $120, and the tele-coach devoted a median 15 min in month 1 plus 10 min monthly thereafter (≈ 2.3 h per participant over 18 months), translating to an amortised equipment cost of US $6.67 and personnel cost of a little money per participant-month—figures compatible with typical oncology-clinic budgets.

## 5 Conclusion

In this prospective cohort, restoring nocturnal melatonin amplitude was associated with a meaningful reduction in tumour-promoting inflammation and, by extension, residual oncogenic risk. Survivors who lifted their night-to-day salivary ratio beyond 2.5, particularly those approaching ≈ 3.0, achieved a mean 0.30-SD reduction in the composite inflammatory score and a 62% lower hazard of recurrence, second primaries, cardiovascular events or death over 18 months; risk declined almost linearly with each 0.2-unit amplitude gain until a plateau near 3.1. Yet the chronobiologic dividend defined here as the aggregate health gain accrued when internal biological clocks are realigned with external time cues was not solely biochemical. Interaction analyses suggested that the protective gradient was most pronounced in stage I–II disease, while advanced stage tempered—but did not abolish—the benefit; joint-exposure models confirmed that the combination of blunted amplitude and persistently high inflammation quadrupled risk relative to rhythm-intact, low-inflammation peers. These observations recast dynamic melatonin profiling and inflammation control as intertwined, modifiable levers for sustaining remission. Bedside salivary assays paired with point-of-care CRP could help triage survivors toward morning bright-light therapy, progressive bedtime advancement and evening blue-light avoidance. Behavioural prescriptions that harness endogenous indoleamine pulses without pharmacologic supplementation. Such behavioural prescriptions harness endogenous indoleamine pulses without pharmacologic supplementation and may represent a low-cost, scalable adjunct to standard survivorship care. Embedding such circadian-hygiene strategies into routine follow-up, reinforced by smartphone-guided light scheduling, may help narrow neuro-immune disparities and support durable recovery as the oesophageal-cancer survivor population continues to grow.

## Data Availability

The original contributions presented in the study are included in the article/supplementary material. Further inquiries can be directed to the corresponding author.
